# Sex, Rhythm & Death: The effect of sexual activity on cardiac arrhythmias and sudden cardiac death

**DOI:** 10.3389/fcvm.2022.987247

**Published:** 2022-09-26

**Authors:** Cicely Anne Dye, Erica Engelstein, Sean Swearingen, Jeanine Murphy, Timothy Larsen, Annabelle Santos Volgman

**Affiliations:** Division of Cardiology, Rush University Medical Center, Chicago, IL, United States

**Keywords:** arrhythmias, sudden cardiac death, sudden cardiac arrest, sex, sexual activity

## Abstract

Arrhythmias and sudden cardiac death with sexual activity are rare. However, the demographics are changing regarding the cardiovascular patients at risk for these events. Recent studies have highlighted that the individuals having cardiac events during sexual activity are becoming younger, with a higher proportion of female decedents than previously described. There needs to be an open dialog between the cardiovascular team and the cardiac patient to provide the education and reassurance necessary for cardiovascular patients to participate in sexual intercourse safely. This paper reviews how sexual activity can lead to an increase in cardiac arrhythmias and sudden cardiac arrest in patients that are not medically optimized or are unaware of their underlying cardiac condition. The most common cardiovascular diseases associated with sexually induced arrhythmias and arrest are discussed regarding their potential risk and the psychosocial impact of this risk on these patients. Finally, cardiovascular medications and implantable cardioverter-defibrillators (ICDs) are addressed by reviewing the literature on the safety profile of these cardiac interventions in this patient population. Overall, sexual activity is safe for most cardiac patients, and providing proper education to the patient and their partner can improve the safety profile for patients with higher risk cardiovascular conditions. To give the appropriate education and reassurance necessary, cardiovascular team members need an understanding of the pathophysiology of how sexual activity can provoke arrhythmias and sudden cardiac arrest. Healthcare providers also need to build comfort in speaking to all patients and ensure that sexual partners, female patients, and those in the LGBTQIA + community receive the same access to counseling but tailored to their individual needs.

## Introduction

Although it is rare, there is considerable trepidation regarding cardiac arrhythmias and sudden cardiac death with sexual activity. This apprehension is not only present in patients with cardiovascular disease but also patients with no known risk factors for arrhythmias or sudden cardiac death. In a recently published study in JAMA, when reviewing 6,847 sudden cardiac deaths across twenty-six years in London, the risk of death during or within an hour of sex was rare (0.2%). However, this study differed from previous studies due to the lower average age of patients at risk and the higher proportion of female decedents (1). While this study was reassuring, it highlights the changing demographic and phenotype of the cardiovascular patient at risk for cardiac arrhythmias and sudden death during sex.

In 2012, the American Heart Association (AHA) published a scientific statement paper on sexual activity and cardiovascular disease (CVD) (2). Despite the overall change in the profile of the cardiovascular patient at risk for arrhythmias and sudden cardiac death during sexual activity, there has not been an AHA update, and this topic is rarely discussed in guidelines (3, 4). As we improve our ability to treat heart disease to decrease morbidity and mortality, we also need to improve awareness and education about how our interventions impact sexual activity in patients at risk for arrhythmias and sudden cardiac death. Sexual activity is a vital component of a patient’s social environment and greatly impacts overall quality of life. Patients with cardiovascular disease and their partners often have questions regarding sexual activity. When these issues are not adequately addressed, depression and anxiety can ensue, which can indirectly worsen clinical outcomes. Clinicians need more knowledge and specific practical training in providing information on sexual matters and counseling patients with various cardiovascular diseases (5–7).

Patients and their partners can have various concerns ranging from sexual performance to concerns about exacerbation of their disease process, which can decrease the frequency of sexual activity or lead to the complete cessation of sexual activity due to fear and anxiety. To explore sexual counseling needs, sexual concerns, and sexual activity of patients with heart failure, a survey study of 45 patients showed that the majority (77%) did not discuss sexual concerns with a health care professional. Sexual concerns included erectile problems (74%), partner overprotectiveness (63%), orgasmic difficulties (51%), lack of sexual interest (42%), and partner fear of sex (36%) (8).

The taboo regarding arrhythmias and sudden cardiac death during sex can extend past the patient and their partner and can also impact clinicians. Health care professionals often feel uncomfortable discussing sexual activity with their patients, particularly female patients and those in the LGBTQIA + community. In a small study of women postmyocardial infarction, many resumed sexual activity without guidance from their physicians. And when a discussion was held about resuming sexual activity the patients usually initiated the conversation (9).

This paper reviews the prevalence and physiology of arrhythmias and sudden cardiac death with sexual activity, and the steps healthcare professionals need to take to provide reassurance while maintaining safety in patients at risk for arrhythmias and sudden cardiac death.

## The pathophysiology of arrhythmias and sudden death during sex

Arrhythmias induced during sexual activity is postulated to occur due to increased sympathetic activation or ischemia due to increased myocardial oxygen demand related to increased physical activity. Patients with channelopathies and inherited cardiomyopathies are more at risk for arrhythmias due to increased sympathetic activation. Whereas those with ischemic heart disease are at increased risk of arrhythmias due to increased myocardial demand during physical activity.

In individuals with channelopathies the pathophysiology on how sympathetic activation precipitates sudden cardiac death varies greatly. In people with Brugada syndrome (BrS) there are multiple theories on the role of the sympathetic nervous system as a potential precipitant of sudden cardiac arrest. These people tend to have reduced cAMP and norepinephrine concentrations found in endomyocardial biopsies, ECG fluctuations with autonomic modulation and a higher incidence of cardiac arrest at night suggesting that cardiac vagal tone may have a significant contribution to arrhythmias in people with BrS (10). Whereas as individuals with Catecholaminergic Polymorphic Ventricular Tachycardia (CPVT) have polymorphic VT from adrenergic stimulation from physical or emotional stressors from an inherited dysfunction in the handling of calcium ions by the sarcoplasmic reticulum in myocardial cells (11). Conversely, Long QT syndrome (LQTS) is a heterogenous group of channelopathies that have genetic mutations associated with cardiac repolarization leading to prolongation of the QT on the surface electrogram that can lead to sudden cardiac death from Torsades de Pointes (TdP). Although there are many precipitants for TdP in patients with LQTS the patients at the greatest risk from risk of SCD with sexual activity are those with LQT1 and LQT2 which can be triggered by physical activity or emotional stress respectively (12).

Inherited cardiomyopathies such as Arrhythmogenic Right Ventricular Cardiomyopathy (ARVC) and Hypertrophic Cardiomyopathy (HCM) can have varied risk profiles in regards to sudden cardiac death based on phenotypic expression, predominant genetic mutation and progression of disease over time. ARVC is an inherited cardiomyopathy that is characterized by the fibrofatty infiltration of the right ventricle but can also infiltrate the left ventricle (13). As this disease progresses patients are at a greater risk for ventricular arrhythmias that can lead to sudden cardiac death. These ventricular arrhythmias are often precipitated by physical exertion and emotional stress. HCM can have a similar clinical presentation with physical activity as a trigger for ventricular arrhythmias and sudden cardiac death, but this cardiomyopathy arises from mutations of the sarcomeric proteins manifesting phenotypically as left ventricular hypertrophy (14).

Finally, ischemic heart disease whether from atherosclerotic heart disease, coronary vasospasm, myocardial bridging or anomalous coronary arteries can cause acute ischemia when there is an increased metabolic demand placed on the heart due to physical activity such as sex which often manifests as chest pain but can rarely present as ventricular fibrillation or polymorphic ventricular tachycardia.

## Sex as a metabolic equivalent and its risk of arrhythmias or sudden cardiac death

**FIGURE 1:**
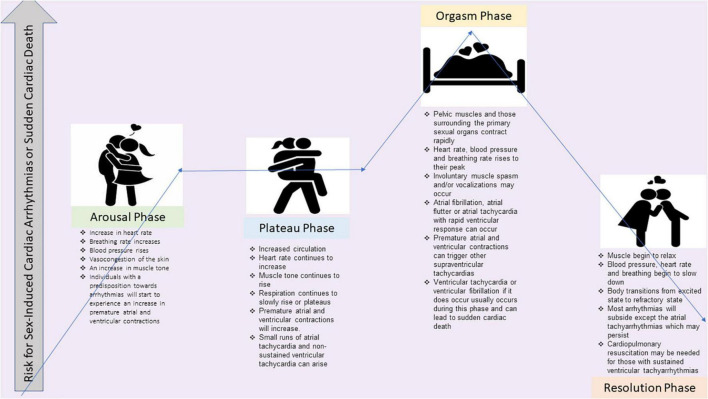
The illustration shows the four phases of sexual intercourse and the potential risk for sex-induced cardiac arrhythmias or sudden cardiac death for each phase. References are Bohlen et al. (15) and Masters and Johnson (16).

Sexual activity is considered to require a low to moderate metabolic equivalent of task (MET), but even mild physical activity in a de-conditioned patient with cardiovascular disease while trying to achieve an orgasm during coitus can precipitate arrhythmias or sudden cardiac death (Figure 1).

When counseling cardiovascular patients, prior to the patient participating in sexual activity, the clinician should ensure that the patient can perform between three to five METs of activity without symptoms. This number is derived from a trial measuring metabolic expenditure in 10 healthy married couples aged 25–43 engaged in foreplay to orgasm and performing various sexual activities and positions during coital and non-coital stimulation by their partner or through self-stimulation (15). If the clinician is unsure from a patient’s history, exercise stress testing can be performed to assess if the patient can achieve three to five METs without symptoms or arrhythmias. Although most patients do not have arrhythmias during sex, in one study, 71% of patients who did experience arrhythmias during near-maximal exercise testing also had arrhythmias during sexual intercourse (15, 16). However, the risk of lethal or hemodynamically significant arrhythmias is rare, with the most reported arrhythmia during sexual activity being ectopic ventricular depolarizations (15, 16).

Most patients with cardiovascular disease can have sex safely without clinical arrhythmias or sudden cardiac death. Even in patients with a known diagnosis of supraventricular tachyarrhythmias, sexual activity is safe and reasonable in well-controlled patients (2). Despite the rare incidence of arrhythmias and sudden cardiac death during sex, some conditions have a higher likelihood of arrhythmias or sudden cardiac death during coitus. This information should be disclosed to the patient and their partner in a reassuring manner that improves safety but diminishes anxiety. The demographic most likely patient to experience sudden cardiac death during coitus is a middle-aged man engaging with a younger partner during an extramarital affair in an unfamiliar setting with a preponderance of ischemic heart disease (17, 18). However, these are not the only patients that benefit from counseling regarding sexual activity. As we improve outcomes in patients with congenital heart disease, channelopathies, non-ischemic structural heart disease and inherited cardiovascular syndromes; counseling regarding sexual activity impacts decreases the likelihood of a rare cardiac in addition to giving these patient the reassurance needed to improve their quality of life and decrease anxiety about childbearing.

## Cardiovascular syndromes at risk for arrhythmias and sudden cardiac death during sex

### Ischemic heart disease

Arrhythmias associated with ischemic heart disease include supraventricular arrhythmias (such as atrial fibrillation) and sudden cardiac death due to ventricular tachycardia and fibrillation. Patients with ischemic heart disease often receive counseling about safely resuming sexual activity to prevent recurrent angina or myocardial infarction; although counseling regarding arrhythmias and the importance of bystander cardiopulmonary resuscitation (CPR) for their partner, while important, is rarely provided. Although sex-associated death is rare and nearly universally witnessed, there is almost a five-fold lower survival rate due to the low rate of bystander CPR (18). Educating a partner on bystander CPR not only has the potential to save the patient’s life but can also decrease fear and anxiety associated with sex and increase the frequency of sexual activity. A review of sexual activity in women after a myocardial infarction showed a reduced desire and decreased frequency of sexual activity due to fear of death during sexual activity (19).

**FIGURE 2:**
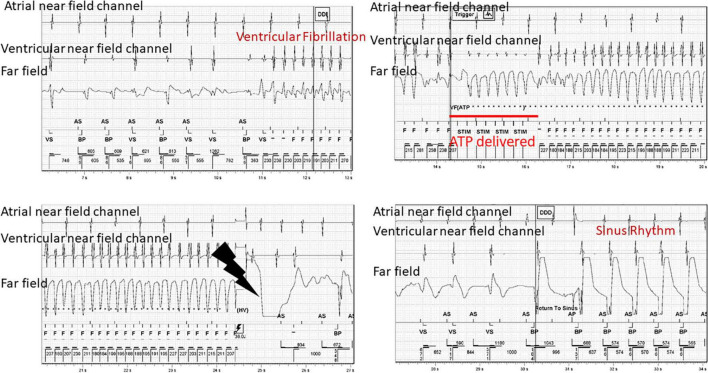
Device tracings of a 70 year old male with ischemic heart disease and prior myocardial infarction who experienced ventricular fibrillation that resulted in delivery of anti-tachycardia pacing and then eventually defibrillation. This event occurred during sexual activity. AS, atrial sensing; VS, ventricular sensing, F, fibrillation zone; STIM, anti-tachycardia pacing.

Patients with ischemic heart disease should also be counseled on using recreational sexual enhancers due to their ability to potentiate arrhythmias. Figure 2 is a tracing of an ICD interrogation of our patient with ischemic heart disease. He used amyl nitrite, also known as “poppers,” which is sometimes used due to its dilatory effects but can also markedly increase heart rate and AV node conduction in these patients leading to tachyarrhythmias such as atrial fibrillation with rapid ventricular rates that can cause pre-syncope or angina during sexual intercourse that can be disconcerting for both the patient and their partner. The tracing shows that his heart rate increased to a heart rate that triggered anti-tachycardia pacing.

### Non-ischemic heart disease and inherited cardiovascular syndromes

Arrhythmias and sudden cardiac death can also occur in patients with non-ischemic structural heart disease or structurally normal hearts, such as those with channelopathies and other inherited cardiovascular syndromes. Non-ischemic structural heart diseases, such as Hypertrophic Cardiomyopathy (HCM), Arrhythmogenic Ventricular Cardiomyopathy (AVC), idiopathic fibrosis, and aortic dissection have been described through registry data and case reports (20). The risk of death during sex in patients with non-ischemic structural heart disease can cause anxiety and depression in these patients and their partners.

In a recent study by Finocchiaro, sex-associated sudden cardiac death was rare and occurred in 17 out of 6,847 cases (0.2%), with 8 of these patients having non-ischemic structural heart disease (1). Similarly, the Paris-SDEC registry (Paris Sudden Cardiac Death Expertise Center) reported that sudden cardiac death due to sex was rare (<1%), but non-ischemic structural heart disease accounted for 12.5% of the sex-related sudden cardiac arrests (18). Overall, patients with non-ischemic heart disease have a very low risk of arrhythmia-related death during sex and can safely participate in sexual activity. Patients and their partners should be made aware of the low risk of arrhythmias and sudden cardiac death during sex but reassured by their cardiologists that these events are rare.

Long QT Syndrome (LQTS) and Catecholaminergic Polymorphic Ventricular Tachycardiac (CPVT) are channelopathies that can lead to arrhythmias such as torsade de pointes (TdP) or polymorphic ventricular tachycardia (PMVT) which can deteriorate into ventricular fibrillation. In an electronic medical review of patients seen by the Genetic Heart Rhythm Clinic, sex-induced cardiac events were more likely in CPVT than in LQTS. Sex-induced cardiac events occurred in two out of forty-three patients (4.7%) with CPVT but in none of the patients with LQTS (21). Despite the low occurrence of sex-related arrhythmias or sudden cardiac death in patients with LQTS, orgasm induced torsades de pointes has been reported in a Long QT Syndrome type 2 (LQT2) patient with a mutation (c.361del) in the KCNH2 gene (chromosome 7q36) (22). Treatment with beta-blockers in these patients can decrease the likelihood of deadly arrhythmias. Proper counseling regarding the safety profile of sex in patients with channelopathies who are well controlled on medications can provide reassurance to the patient and their sexual partner.

## Sex in patients with cardiomyopathies with implantable cardioverter- defibrillators and on goal directed medical therapy

ICDs are implanted in patients as either primary or secondary prevention of life-threatening arrhythmias (ventricular tachycardia and fibrillation). Although ICDs are life-saving devices for at-risk patients, receiving a shock during sexual activity or fear that their partner might receive an ICD shock is a frequent concern of patients and their partners. It causes significant anxiety and fear of sexual activity (23, 24). Counseling before and after device implantation can decrease the anxiety in patients requiring ICDs. According to the AHA Scientific Statement on Sexual Activity and Cardiovascular Disease, sexual activity is reasonable in patients with ICDs for primary prevention and in those requiring secondary prevention that can perform three to five METs without precipitating VT or VF. Sexual activity should be deferred in patients who have received multiple shocks until the causative arrhythmia has been stabilized and they can safely perform three to five METs without arrhythmias (2).

Optimizing patients so that they can perform three to five METs so that they can participate in sexual activity can be achieved through cardiac rehab. A recent meta-analysis of fourteen trials showed that cardiac rehabilitation could improve sexual function (25). In addition to cardiac rehabilitation, improving the arrhythmia burden through ablations and goal-directed medical therapy can also help patients improve their physical activity levels. Unfortunately, despite best medical practices, sexual dysfunction can still arise due to a patient’s underlying disease process and the prescribed cardiovascular medications.

**FIGURE 3:**
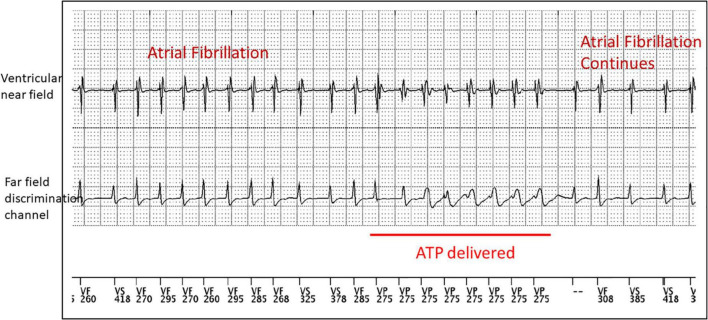
Device tracings of a 50 year old male with non-ischemic heart disease who experienced episodes of atrial fibrillation with rapid AV conduction into his ventricular fibrillation zone that resulted in the delivery of anti-tachycardia pacing. This event occurred while taking amyl nitrite during sexual activity. ATP, anti-tachycardia pacing.

Cardiovascular medications can cause erectile dysfunction, decreased sex drive, and decreased vaginal lubrication due to their mechanism of action and the nocebo effect (26, 27). This can contribute to patients discontinuing their cardiac medications without disclosing to their provider due to embarrassment, leading to poorly controlled cardiac arrhythmias and inappropriate shocks (Figure 3) and in the worst-case scenario sudden cardiac death.

Counseling, education, and shared decision-making can improve patient adherence to cardiac medications. If the patient continues to have significant issues regarding sex drive, erectile dysfunction, or vaginal dryness, alternate but equivalent medications can be prescribed. For patients with cardiovascular disease and erectile dysfunction, phosphodiesterase type 5 inhibitors can be prescribed in patients not taking long-acting nitrates. Vaginal dryness can be safely treated with topical estrogens to decrease pain with sexual intercourse (26, 27). Finally, individual and couple’s therapy can also be an effective adjunct to improving sex drive for patients with cardiomyopathies and ICDs.

## Conclusion

Sex is a salient part of life and procreation that contributes to overall wellness and quality of life. In most individuals, sex can occur safely without risk of death or arrhythmias. But it is devastating when arrhythmias or sudden cardiac death do occur during sexual activity. Patients and their partners should be given reassurance that sex is reasonable and safe in most situations. However, they should also receive personalized counseling and education on the likelihood of arrhythmias or sudden cardiac death as it applies to their disease process and how to minimize these events with medication adherence, avoiding non-FDA-approved sexual enhancers, and the importance of bystander CPR.

Medical providers can improve outcomes in patients at risk for arrhythmias and sudden cardiac death by initiating a conversation about sexual activity with their at-risk patients. During these counseling sessions, the provider should listen to the patient’s concerns regarding sexual activity and make shared decisions regarding cardiovascular medications and devices while abiding by current medical guidelines. Cardiovascular specialists can help stratify patients at risk for arrhythmias and sudden cardiac death by utilizing exercise stress tests and improve outcomes by enrolling patients in cardiac rehabilitation to improve aerobic conditioning when appropriate. Health care professionals also need to be aware of potential disparities in healthcare and ensure that sexual partners, female patients, and those in the LGBTQIA + community receive the same access to counseling but tailored to their individual needs.

## Author contributions

**FIGURE 4:**
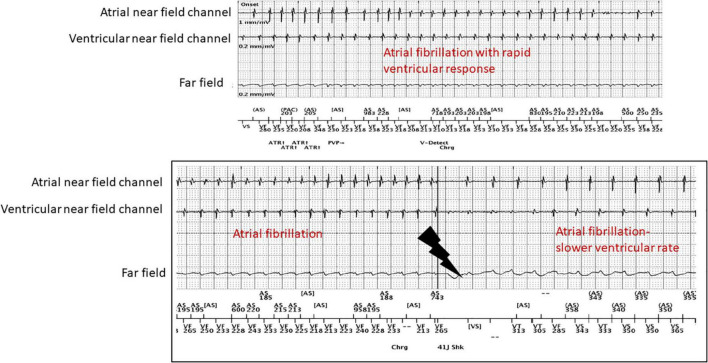
Patient with atrial fibrillation with rapid ventricular response with an inappropriate shock after stopping his beta-blocker due to concerns for erectile dysfunction.

CD and AV created the outline, wrote sections, and edited the entire manuscript. CD created Figure 1. CD, TL, and JM created Figures 2–4. EE and SS wrote several sections. TL edited the entire manuscript. All authors contributed to the article and approved the submitted version.
